# Efficacy of NEMO-binding domain peptide used to treat experimental osteomyelitis caused by methicillin-resistant *Staphylococcus aureus*: an in-vivo study

**DOI:** 10.1186/s13756-019-0627-y

**Published:** 2019-11-20

**Authors:** Chang-Peng Xu, Ya Chen, Hong-Tao Sun, Zhuang Cui, Ya-Jun Yang, Lei Huang, Bin Yu, Fa-Zheng Wang, Qing-Po Yang, Yong Qi

**Affiliations:** 1 Department of Orthopaedics, Guangdong Second Provincial General Hospital, NO.466 Xingang Road, Haizhu District, Guangzhou, 510317 People’s Republic of China; 2 Department of Orthopaedics and Traumatology, Nanfang Hospital, Southern Medical University, Guangzhou, Guangdong People’s Republic of China; 3 Department of Pathology, Guangdong Medical University, Zhanjiang, Guangdong People’s Republic of China; 4 Department of Orthopaedics, The First People’s Hospital of Kashgar Prefecture, Kashgar, Xinjiang People’s Republic of China

**Keywords:** NBD peptide, Osteomyelitis, Methicillin-resistant *Staphylococcus aureus*, Vancomycin, Rabbit

## Abstract

**Purpose:**

Treatment of chronic osteomyelitis (bone infection) remains a clinical challenge. Our previous study had demonstrated that NEMO-binding domain (NBD) peptide effectively ameliorates the inhibition of osteoblast differentiation by TNF-α in vitro. In this work, NBD peptide was evaluated in vivo for treating chronic osteomyelitis induced by methicillin-resistant *Staphylococcus aureus* (MRSA) in a rabbit model.

**Methods:**

Tibial osteomyelitis was induced in 50 New Zealand white rabbits by tibial canal inoculation of MRSA strain. After 3 weeks, 45 rabbits with osteomyelitis were randomly divided into four groups that correspondingly received the following interventions: 1) Control group (9 rabbits, no treatment); 2) Van group (12 rabbits, debridement and parenteral treatment with vancomycin); 3) NBD + Van group (12 rabbits, debridement and local NBD peptide injection, plus parenteral treatment with vancomycin); 4) NBD group (12 rabbits, debridement and local NBD peptide injection). Blood samples were collected weekly for the measurement of leucocyte count, erythrocyte sedimentation rate (ESR), and C-reactive protein (CRP) levels. The rabbits in all four groups were sacrificed 6 weeks after debridement; the anti-infective efficacy was evaluated by radiological, histological, and microbiological examination, and promotion of bone remodeling was quantified by micro-CT using the newly formed bone.

**Results:**

Except two rabbits in the Control group and one in the NBD group that died from severe infection before the end point, the remaining 42 animals (7, 12, 12, 11 in the Control, Van, NBD + Van, and NBD group respectively) were sacrificed 6 weeks after debridement. In general, there was no significant difference in the leucocyte count, and ESR and CRP levels, although there were fluctuations throughout the follow-up period after debridement. MRSA was still detectable in bone tissue samples of all animals. Interestingly, treatment with NBD peptide plus vancomycin significantly reduced radiological and histological severity scores compared to that in other groups. The best therapeutic efficacy in bone defect repair was observed in the NBD peptide + Van group.

**Conclusions:**

In a model of osteomyelitis induced by MRSA, despite the failure in demonstrating antibacterial effectiveness of NBD peptide in vivo, the results suggest antibiotics in conjunction with NBD peptide to possibly have promising therapeutic potential in osteomyelitis.

## Introduction

Chronic osteomyelitis is a bone infection with hallmarks of progressive bone necrosis and sequestrum formation [1]. Due to the recurrence and chronicity of osteomyelitis, it is often necessary that the infected bone be debrided, tissues reconstructed, and long-term antibiotic therapy administered [2]. The Gram-positive organism *Staphylococcus aureus (S. aureus)* is the most common causative agent of osteomyelitis, accounting for approximately 80% of all human cases [3]. The growing incidence of antibiotic-resistant *S. aureus* strains can explain the recurrent attacks of osteomyelitis in patients undergoing therapy [4]. To encounter the multidrug-resistance challenge, developed from the systemic antibiotic usage, local antibiotic delivery including polymethylmethacrylate (PMMA) cement and biodegradable materials has been introduced, which increases the local antibiotic concentration and simultaneously minimizes their systemic toxicity [5]. However, the cement may act as a foreign body when antibiotics are no longer being released, requiring additional surgery for its removal. In addition, finding new antibiotic substances for antibiotic resistance is expensive and often involves compromised efficacy within short time periods due to the enormous potential of rapid adaptation in microorganisms. Recently, “cellular hysteresis” strategy, using the currently available antibiotics, was harnessed to optimize the antibiotic therapy, in order to achieve both enhanced elimination of bacteria and reduced evolution of resistance [6].

*S. aureus* is a capable bone pathogen with adhesion molecules that facilitate its binding to the bone matrix and toxin secretion, thereby stimulating bone resorption [7]. Proinflammatory cytokines such as interleukin 1 (IL-1), interleukin 6 (IL-6), or tumor necrosis factor alpha (TNF-α), are produced in *S. aureus*-induced osteomyelitis [8]. *S. aureus* may also activate the nuclear factor kappa B (NF-κB) pathway and ensure that NF-κB activation is required for the phagocytosis of *S. aureus* by macrophages [9]. TNF-α acts on marrow-derived macrophages to promote the induction of differentiation to osteoclast cells, and the receptor activator of nuclear factor kappa B ligand (RANKL) acts on mature osteoclast cells to induce bone resorption activity [10]. Our group and Yamazaki et al. [11, 12] had previously shown that TNF-α inhibits osteoblast differentiation through the activation of NF-κB, which directly leads to the abrogation of Smad1 signaling to hamper bone formation activity. Taken together, NF-κB seems to be a critical molecular switch for several downstream events that affect host responses to bone infection in osteomyelitis.

Drugs that selectively target inflammation-induced NF-κB activity while sparing the protective functions of basal NF-κB levels would be of greater therapeutic value and would likely display fewer undesired side-effects. Phosphorylation of IκB proteins by the IκB kinase (IKK) complex is a crucial step in pathways leading to NF-κB activation. The IKK complex contains two catalytic subunits (IKKα and IKKβ) and a regulatory component named NF-κB essential modulator (NEMO) or IKKγ [13]. A short cell-permeable peptide spanning the NEMO-binding domain (NBD) disrupts the association of NEMO with IKKβ, blocks TNF-α-induced NF-κB activation in vitro, and effectively ameliorates responses in distinct animal models of inflammation [14]. Recent identification and characterization of NBD peptide have provided an opportunity to selectively abrogate the inflammation-induced activation of NF-κB by targeting the NBD-NEMO interaction [15]. A previous study [11] in our laboratory had shown that application of NBD peptide could ameliorate the osteoblast differentiation inhibition caused by TNF-α-induced NF-κB activation. Taken together, we hypothesized that NBD peptide could be a promising tool for osteomyelitis treatment, owing to its usefulness in controlling inflammatory bone resorption and accelerating bone regeneration. The present study aimed to investigate the efficacy of NBD peptide, based on NF-κB blockade, in inflammation suppression and bone regeneration using a rabbit model of osteomyelits.

## Materials and methods

### Animals

Approval was obtained from Institutional Animal Care and Use Committee prior to performing this study. All animals were treated according to the guidelines for laboratory animal treatment and care, and all protocols were approved by the local animal welfare committee. Fifty healthy, pathogen-free adult New Zealand white rabbits (weighing 2.42–2.76 kg) were chosen for the experiment; they were individually caged, fed a standard balanced chow, and provided with water ad libitum.

### NBD peptide synthesis

A 23-amino acid cell-permeable NBD peptide (YGRKKRRQRRR-G-TTLDWSWLQME) was synthesized, and purified by high performance liquid chromatography, according to our previous study [11].

### Osteomyelitis model induction

The rabbit model of chronic osteomyelitis was induced as described in Nijhof et al. [16]. Rabbit weights were recorded, and ketamine (35 mg/kg) and xylazine (5 mg/kg), for anesthesia, were injected subcutaneously under strictly aseptic conditions. The operation area of proximal right tibia was shaved, disinfected with povidone-iodine, and covered by sterile drapes, before injecting lidocaine (5 mg/kg) for local anesthesia. After making a 2.0-cm skin incision on the anterolateral surface of the right proximal tibia, the cortex of metaphysis was partially exposed. A hole was drilled in the cortex using a 2.0-mm Kirschner wire, saline irrigated, and bone marrow extracted by means of 18-gauge needle, which was inserted into the medullary canal. Sequentially, 0.1 ml of 5% sodium morrhuate, 0.1 ml of bacterial suspension of MRSA, ATCC 43300 (1 × 10^6^ CFU/ml), and 0.1 ml of saline (0.9%) were injected into the medullary cavity. After sealing the holes with sterile bone wax to prevent leakage of the injection, the fascia, subcutaneous layer, and skin were irrigated and closed with 4/0 vicryl. General conditions, including food and water intake, activity, and presence of localized and systemic infections, were monitored daily for 3 weeks after initial operation. There was no death of rabbits during the follow-up period, and standard anteroposterior- and lateral-view radiographs were taken for all animals, at 3 weeks post-operation, to determine the severity of osteomyelitis according to the parameters for osteomyelitis described by Norden et al. [17]. Meanwhile, five parameters, including periosteal new bone, bone destruction, sequestra, soft tissue calcification, and soft tissue swelling, were determined for each tibia, and animals with three points or more were diagnosed with osteomyelitis.

### Treatment groups

Forty-five animals were finally diagnosed with osteomyelitis and randomly divided into four groups: Control, Van, NBD, and NBD + Van groups. Animals in the Control group (*n* = 9) were left untreated. Animals in the Van group (*n* = 12) were treated with debridement and intravenous injection of vancomycin (30 mg/kg, twice daily) for 4 weeks. Animals in the NBD group (*n* = 12) were treated with debridement and local percutaneous injection of NBD peptides (5 mg/kg BW) into the medullary cavity (immediately after debridement, 2 and 4 weeks post-operation). Animals in the NBD + Van group (n = 12) received the same treatment as the Van group, plus local injection with NBD peptides as the NBD group. The post-operative follow-up after debridement lasted for 6 weeks, during which the animals were housed in individual cages and fed a normal diet.

For the debridement procedure, an 8 mm × 20 mm cortical bone window in the anteromedial surface of right proximal tibia was generated to clean out all infectious and necrotic tissues, according to the varying radiological severity of osteomyelitis. Before wound closure, the NBD peptides were injected into the medullary cavity of animals in the NBD + Van and NBD groups. Finally, the removed samples were sent for a culture study.

### Laboratory monitoring

After debridement procedure, all animals in the four groups were followed closely for any clinical sign of septic spread. Blood samples were collected weekly for the measurement of leucocyte count, erythrocyte sedimentation rate (ESR), and C-reactive protein (CRP) levels.

### Radiological, microbiological, and histological examination

After the debridement procedure, standard anteroposterior- and lateral-view radiographs were taken on day 1, and 3 and 6 weeks post-operation, for all animals. According to the severity scoring system for osteomyelitis, described by Norden et al., all the radiographs were assessed in a blinded fashion by an orthopedic surgeon.

At 6 weeks after debridement, the animals were euthanized by an intravenous injection of excess pentobarbital sodium. In the same way as the first operation, the tibiae were excised and freed from soft tissues under aseptic conditions. With sterile curettes, and swabbing the initial bone window, samples were obtained and sent for a culture study to analyze the infection rates.

Tibia samples were used for micro-computed tomography. Morphology of the reconstructed tibial cortex was assessed using an animal micro-CT scanner (eXplore Locus, GE Healthcare Biosciences, London, UK). Briefly, the specimens were scanned with a 55 kVp energy setting, having intensity of 145 mA with 200 ms acquisition time, and no frame average in high-resolution mode, which provided a voxel resolution of 12 μm. After micro-CT scan, the defect region was identified by a contour as a traced region of interest (ROI), and the relative measurements were calculated, including bone mineral densities (BMDs) and bone volume/total volume (BV/TV).

Next, conventional pathological sections stained with hematoxylin and eosin were prepared for bone samples, and decalcified cross sections (5-μm) were assessed by a pathologist in a blinded fashion according to the scoring system of Smeltzer et al. [18]. Animals were diagnosed with osteomyelitis when they possessed four or more points.

### Statistical analysis

The Statistical Package for the Social Sciences (SPSS) (SPSS Inc., Chicago, IL) version 11.0 for Windows was used to analyze the results. Mann-Whitney tests for independent samples were performed to compare the laboratory values and radiological and histological scores across the groups. Newly formed bone volume was compared by analysis of variance. Fisher’s exact test was used to compare infection rates across the groups. Unless stated otherwise, results are presented as mean ± standard deviation, and performed in triplicate; *p*-values of 0.05 or less were considered to be significant.

## Results

### General conditions

**Fig. 1:**
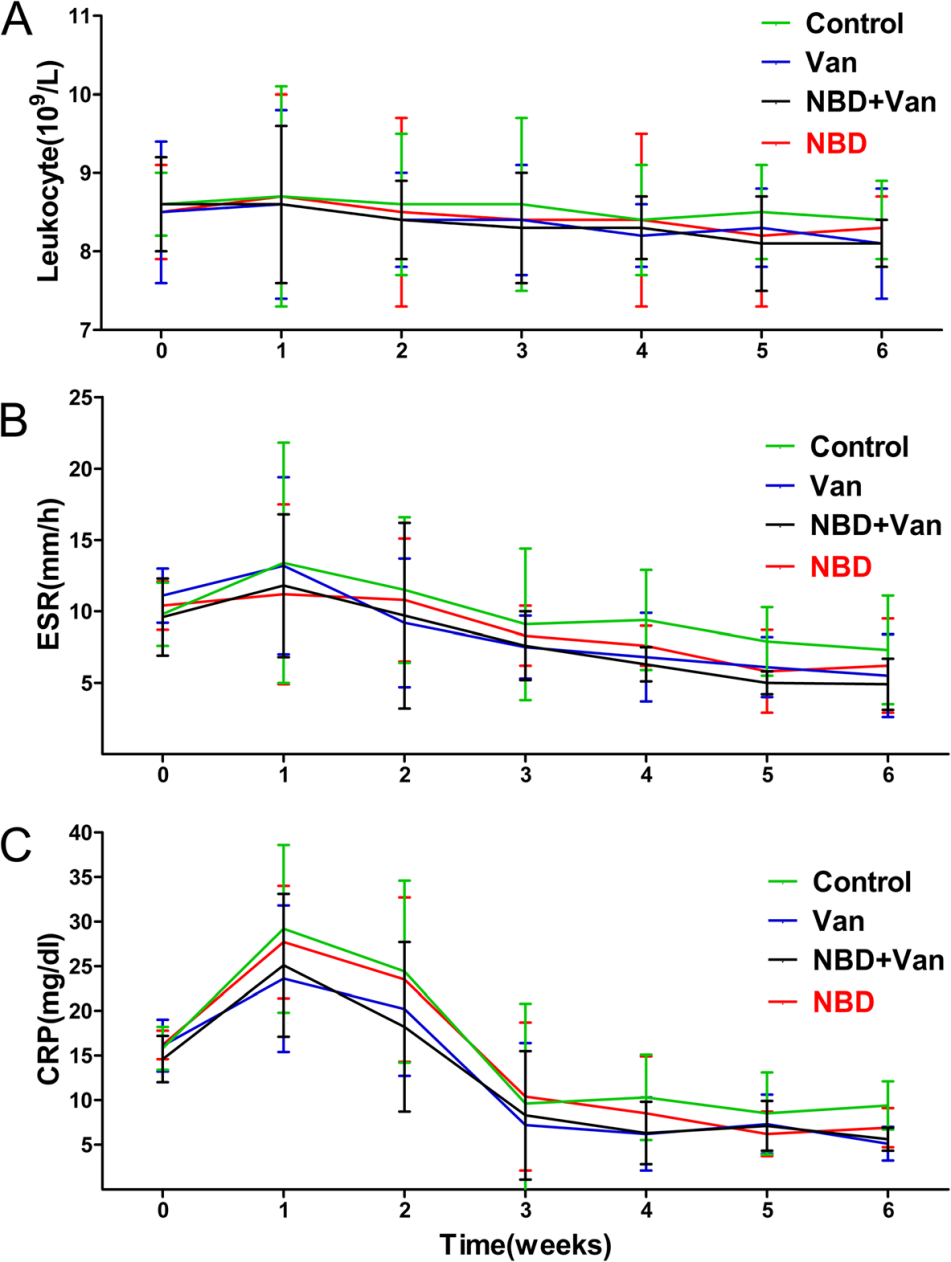
Leucocyte count (**a**), and ESR (**b**) and CRP (**c**) levels in the Control, Van, NBD + Van, and NBD groups during the 6 weeks follow-up period after debridement

The clinical signs of infection varied from mild to severe, following induction, and in the one-week follow-up after debridement, two rabbits in the Control group and one in the NBD group died from severe infection, as confirmed by autopsy. The remaining 42 animals recovered well, with no obvious sign of systemic complication in the 6-week follow-up, and were included in statistical analysis. Furthermore, laboratory analyses did not reveal any significant change of leucocyte count, and ESR and CRP levels (Fig. 1), although there were fluctuations throughout the follow-up period after debridement.

### Microbiology

**Fig. 2:**
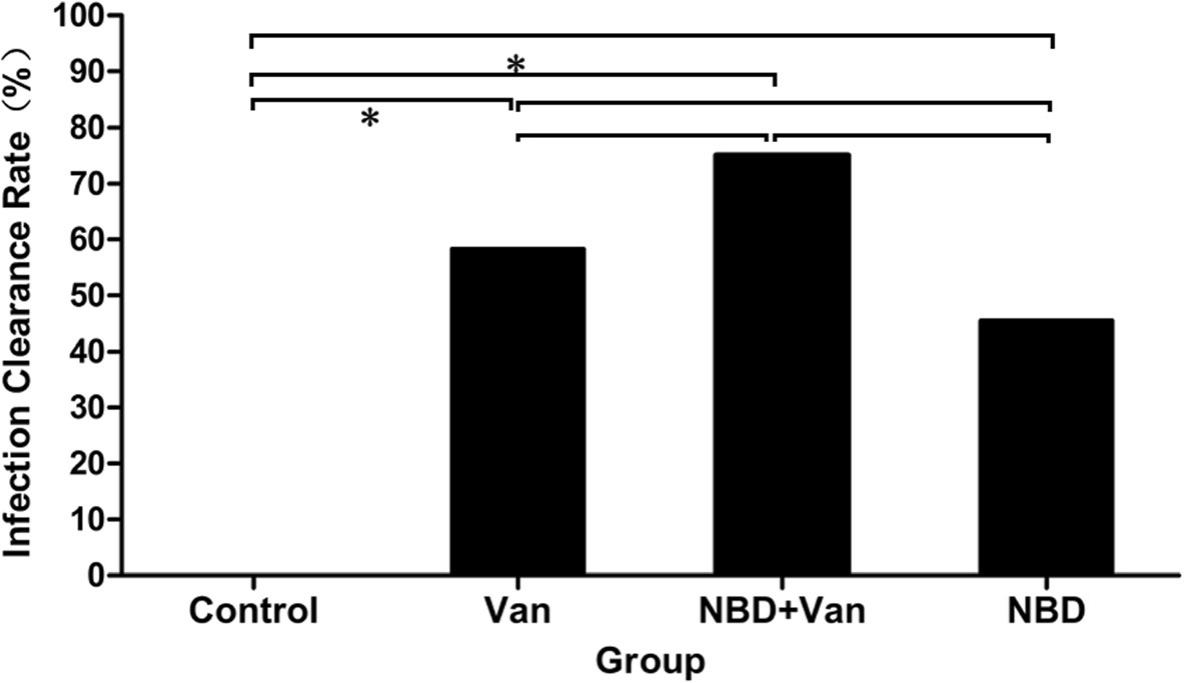
Infection clearance rates of MRSA in the Control, Van, NBD + Van, and NBD groups at 6 weeks after debridement. The Control group showed the lowest infection clearance rate, while the highest infection clearance rate was detected in the NBD + Van group. Fisher’s exact test determined statistical significance where *p < 0.05

While performing debridement, the culture of curetted samples showed all 42 rabbits to have MRSA. At sacrifice, 7 of 7, 5 of 12, 3 of 12, and 6 of 11 animals in the Control, Van, NBD + Van, and NBD groups, respectively, were tested positive for MRSA (Fig. 2). Infection clearance rate of the Control group was significantly lower than that of the Van (*P* = 0.017) and NBD + Van groups (*P* = 0.003). Infection clearance rate of the Control group was also lower than that of the NBD group, although it was not significant (*P* = 0.101). NBD + Van group showed the highest infection clearance rate, although there was no significant difference with the Van (*P* = 0.667) or NBD group (*P* = 0.214). Infection clearance rate of the NBD group was a little lower than that of the Van group, although the difference was not significant (*P* = 0.684). Altogether, there was no significant difference among the treatment groups (Van, NBD + Van, and NBD) with regard to infection clearance rate, although the best therapeutic efficacy was confirmed by the optimal infection elimination in the NBD peptide + Van group.

### Radiography

**Fig. 3:**
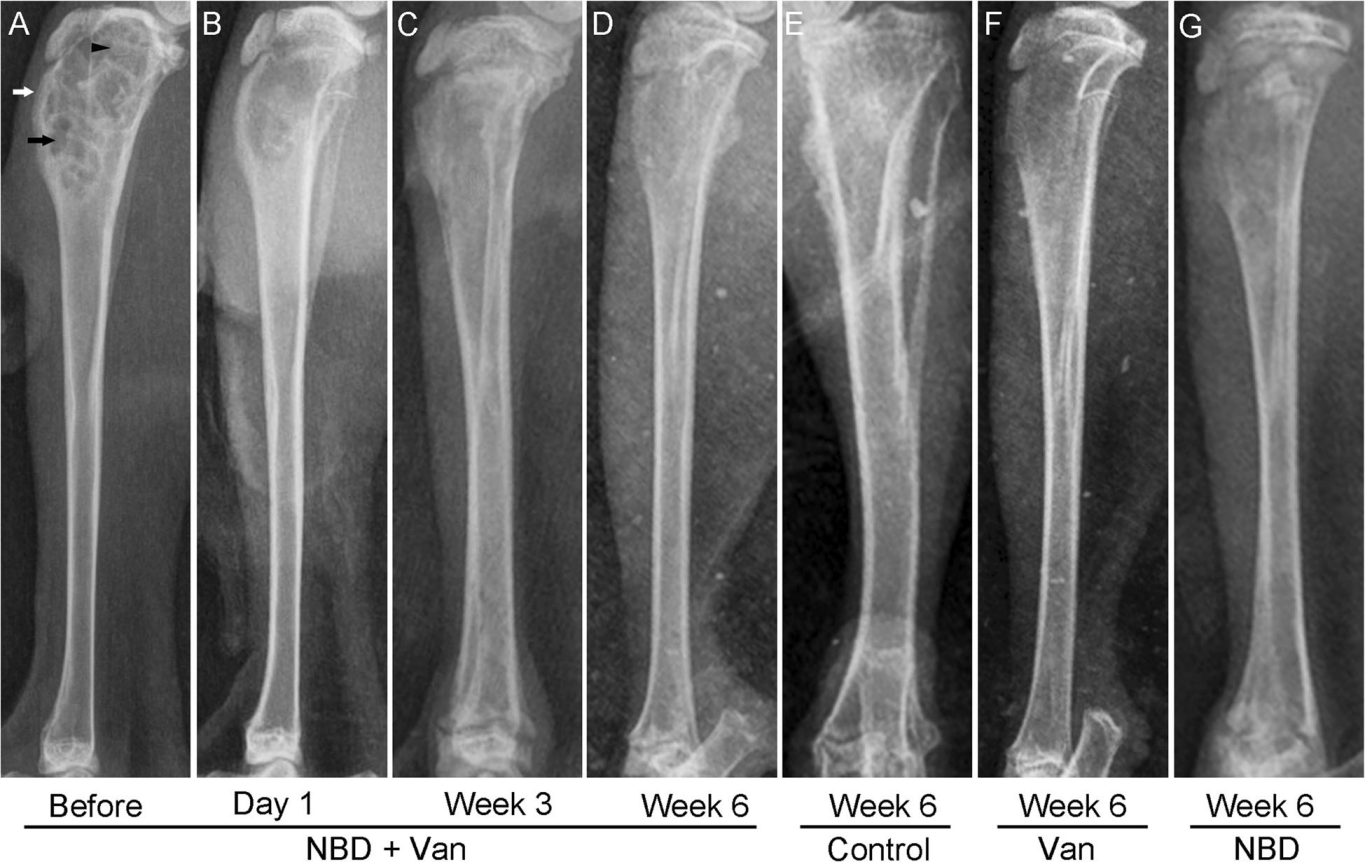
Lateral radiographs of tibiae in different groups, taken before and 1 day, 3 and 6 weeks after debridement. **a** 3 weeks after infection induction, the tibia showed a typical character of chronic osteomyelitis, including periosteal new bone formation (white arrow), bone destruction (black arrow), and sequestra (black arrows). The tibiae in the NBD + Van group have shown a gradually healing of osteomyelitis after treatment (**b**, **c**, and **d**), whereas the tibial osteomyelitis in other groups did not ameliorate obviously (**e**, **f**, and **g**)

**Fig. 4:**
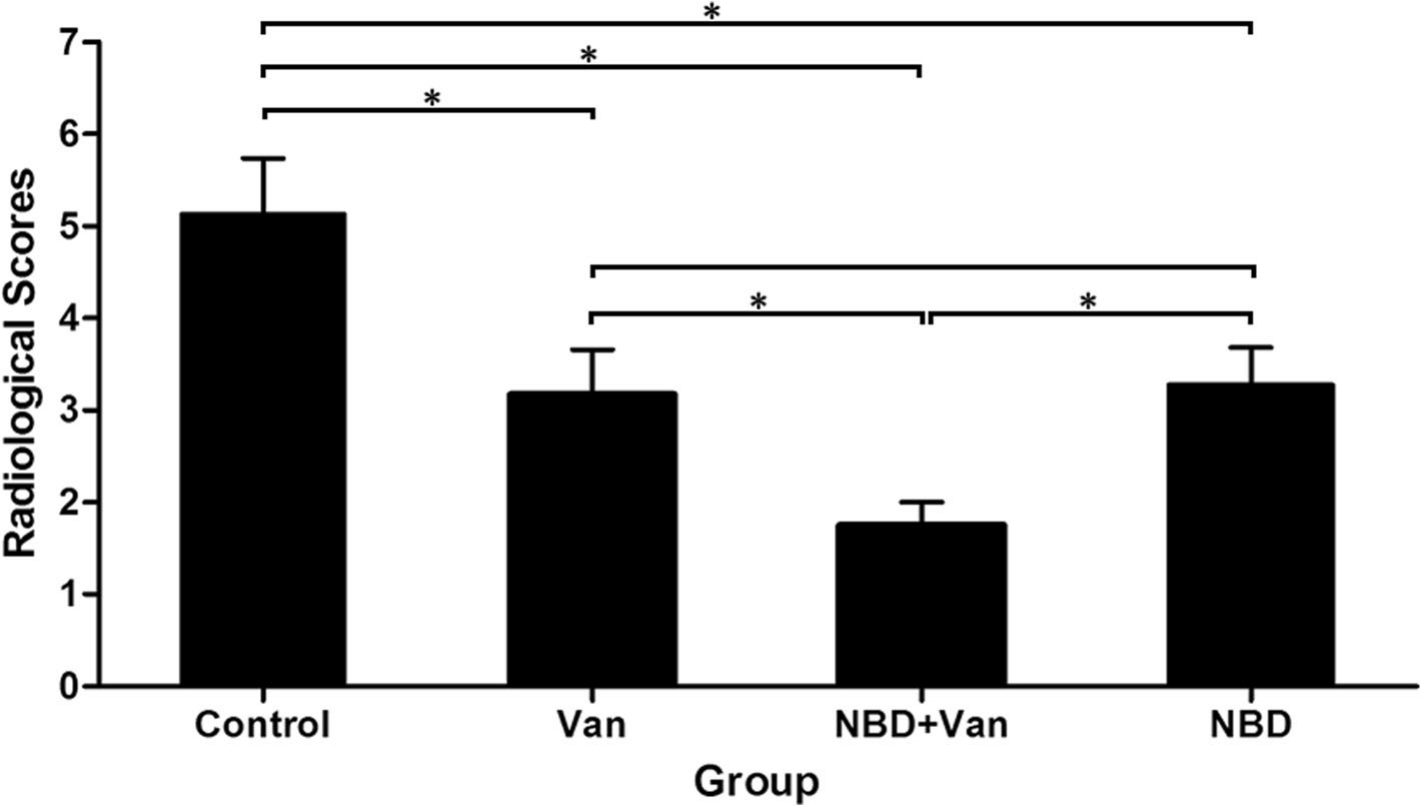
Radiological scores in the Control, Van, NBD + Van, and NBD groups 6 weeks after debridement. Results were shown as mean ± standard deviation. Mann-Whitney test determined statistical significance where * *P* < 0.05

At 3 weeks after infection introduction, typical signs of bone infection, including periosteal new bone, bone destruction, and sequestra, could be seen in the radiographs of tibiae (Fig. 3a). After treatment for 6 weeks, elimination of bone infection and bone window healing were observed in the NBD + Van group (Fig. 3b–d), with only 3 of 12 animals having a score of three or above. Different degrees of infection were observed in the Control, Van, and NBD groups (Fig. 3e–g), with 7 of 7, 5 of 12, and 6 of 11 animals having a score of three or above, respectively. Total radiological score of the Control group was significantly higher than that of the Van (*P* = 0.031), NBD + Van (*P* = 0.001), and NBD groups (*P* = 0.03). The NBD + Van group showed a significantly lower score than the Van (*P* = 0.03) and NBD groups (*P* = 0.007). No statistically significant difference was found between the Van and NBD groups (*P* = 0.767) (Fig. 4)**.**

**Fig. 5:**
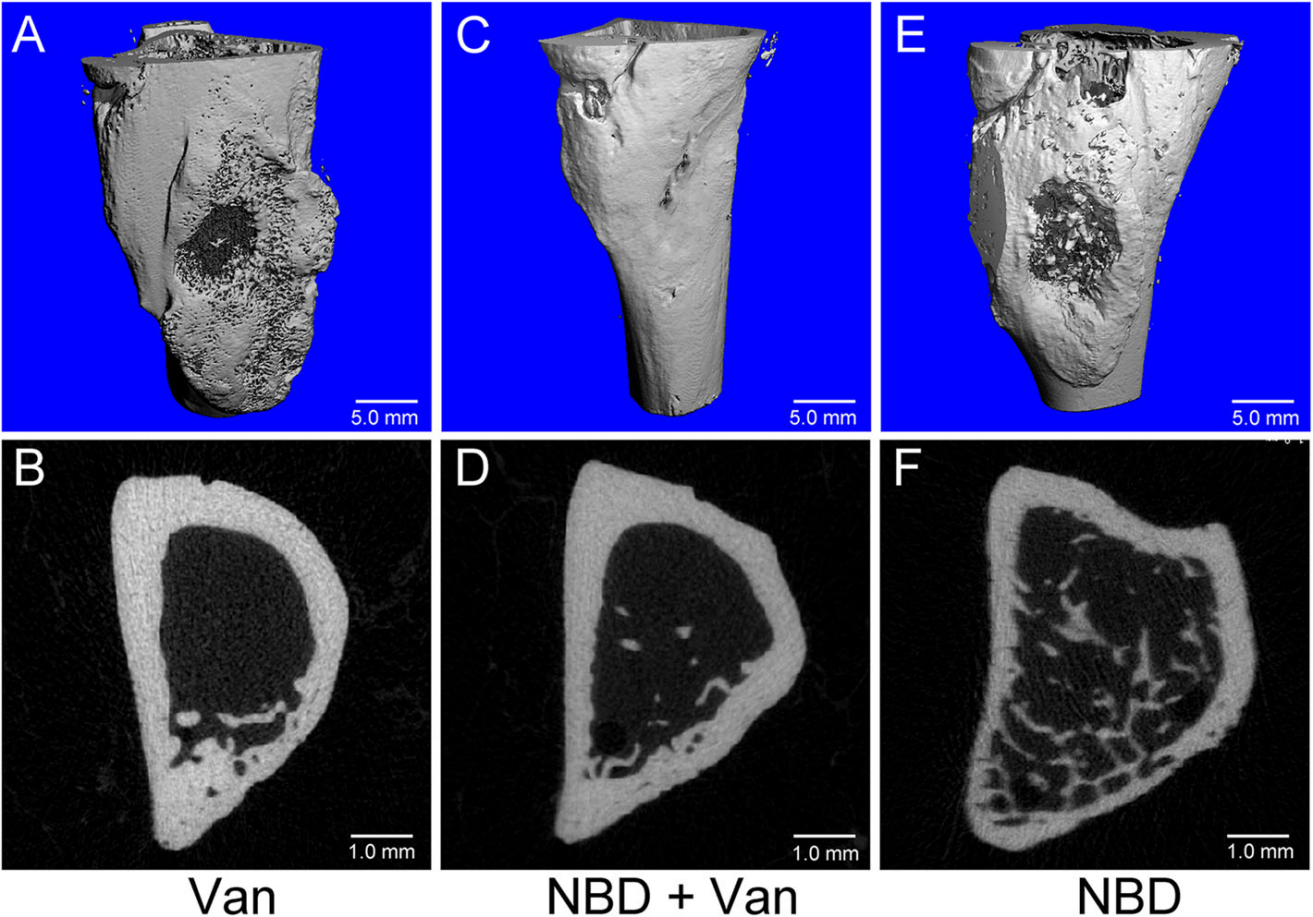
Representative 3D micro-CT reconstruction and Micro-CT cross sections analysis of the proximal tibia in the Van (**a**, **b**), NBD + Van (**c**, **d**), and NBD (**e**, **f**) group 6 weeks after debridement. The bone window in the NBD + Van group was covered by newly formed bone, while no sufficient bone formed in other two groups

As shown in Fig. 5, morphology of the newly formed bone was reconstructed using micro-CT in the Van, NBD + Van, and NBD groups (Fig. 5a, c, e), and the results were almost consistent with the x-ray images. Micro-CT cross sections of the defect area showed more obvious new bone formation in the NBD + Van group (Fig. 5d). Further, there were several cavities scattered over the area in the Van and NBD groups (Fig. 5b, f). Compared to the NBD group, Van group exhibited greater new bone formation, although the cortex was incomplete and there were voids beneath the cortex. Compared to the Van group, NBD + Van group showed improved new cortex formation. In all groups, sporadic trabecular bone was observed in the marrow canal.

**Fig. 6:**
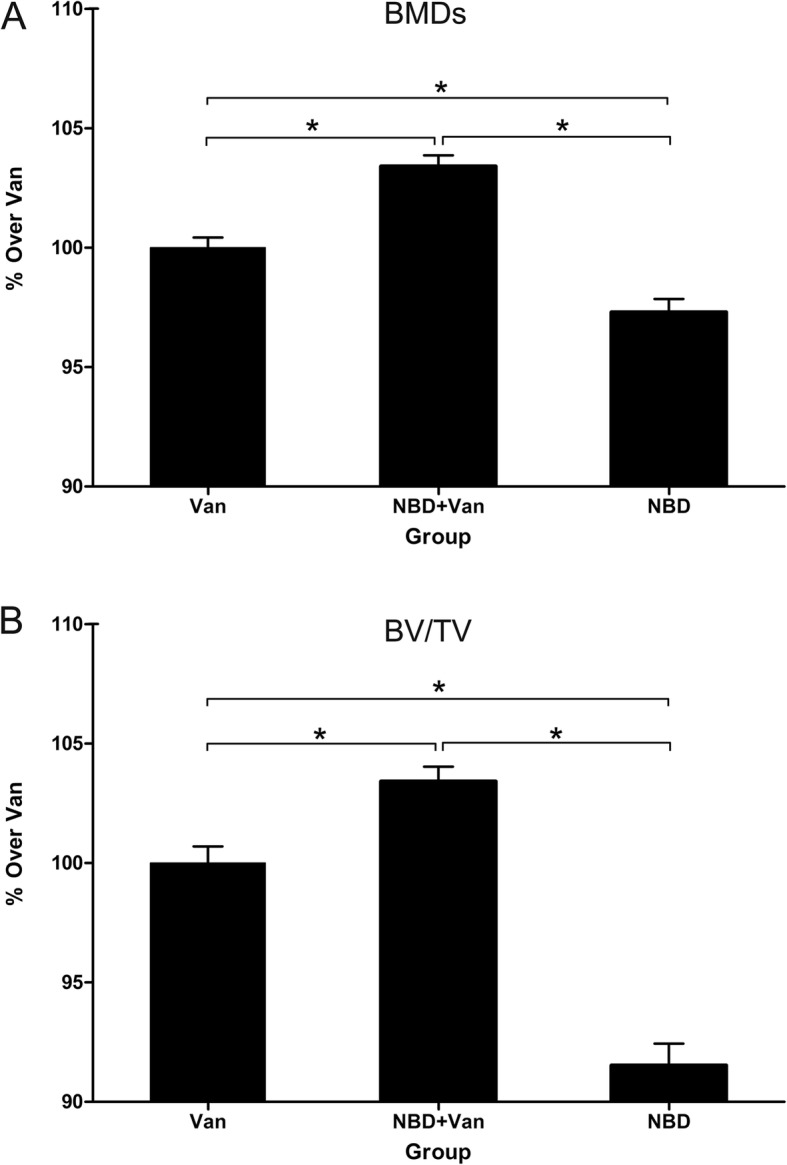
Morphometric evaluation of the local bone mineral densities (BMDs) (**a**) in the new bone formation area and bone volume/total volume (BV/TV) (**b**) in the Van, NBD + Van, and NBD groups 6 weeks after debridement. Results were shown as mean ± standard deviation. Analysis of variance test determined statistical significance where * *P* < 0.05

The amount of newly formed bone in the defect sites was calculated by morphometric analysis (Fig. 6). The NBD group exhibited the lowest levels of both BMDs and BV/TV at the defect site when compared to other groups (*P* = 0.001). BMDs at the defect site of NBD + Van group were significantly higher than that of the Van and NBD groups (*P* = 0.001 and *P* = 0.001, respectively). The BV/TV results exhibited a similar trend as the BMD data. Mean BV/TV percentage in the NBD + Van group was significantly higher than that in the Van and NBD groups (*P* = 0.001); there was also a significant difference in BV/TV between the Van and NBD groups (*P* = 0.001).

### Histology

**Fig. 7:**
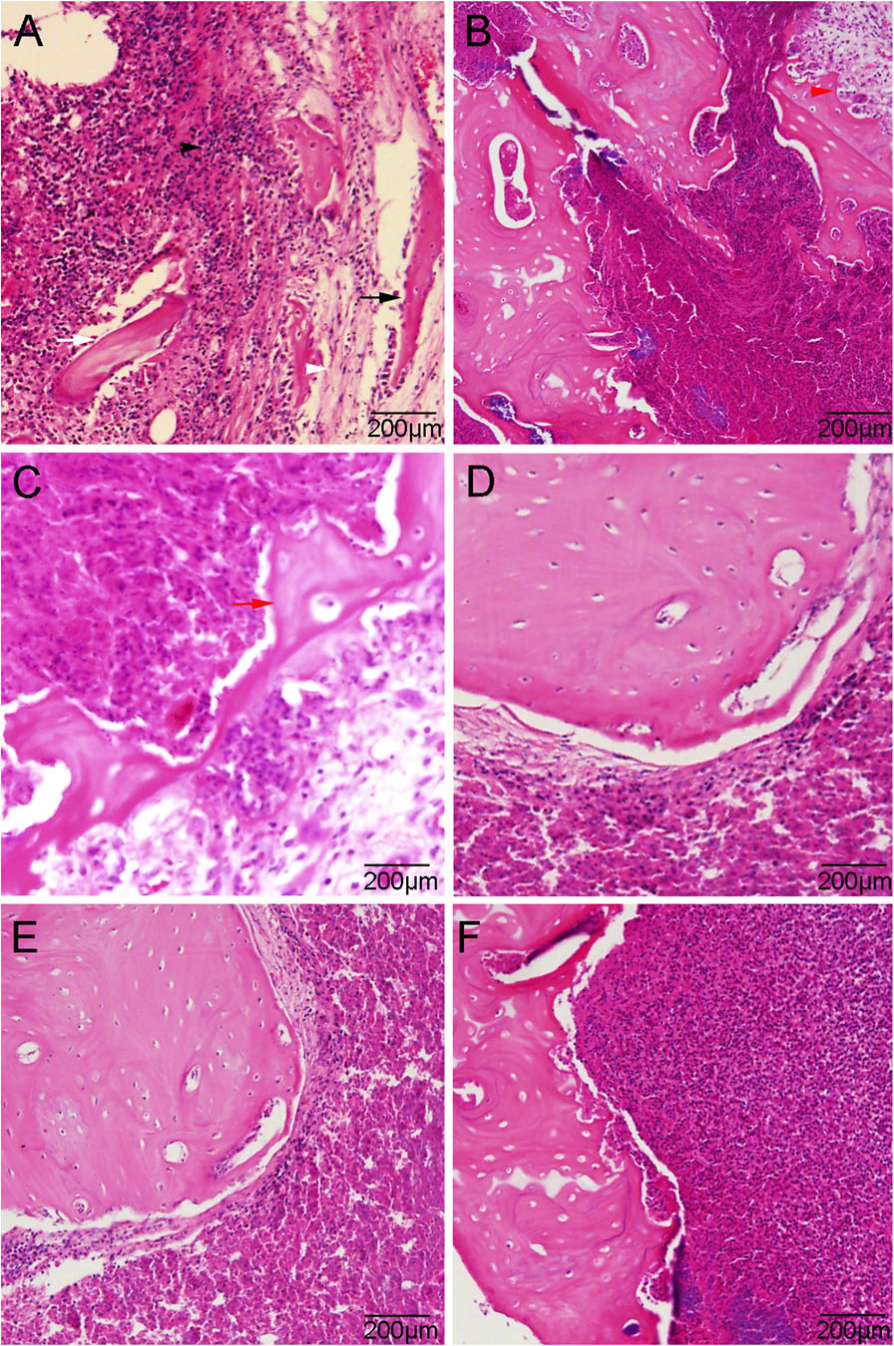
Photomicrographs of the control group (**a** and **b**) shows destruction of cortical bone (black arrow), sequestrum formation (white arrow), intramedullary abscess (black arrow-head), fibrosis (white arrow-head), and proliferated foamy histotcytes (red arrow-head). The van group (**c**) and NBD group (**d**) showed relatively moderate osteomyelitis characteristics, with some new bone formation (red arrow). Photomicrographs of the NBD + Van group (**e** and **f**) were shown mild destruction of cortical bone and intramedullary new bone formation, without signs of severe inflammation (haematoxylin and eosin)

**Fig. 8:**
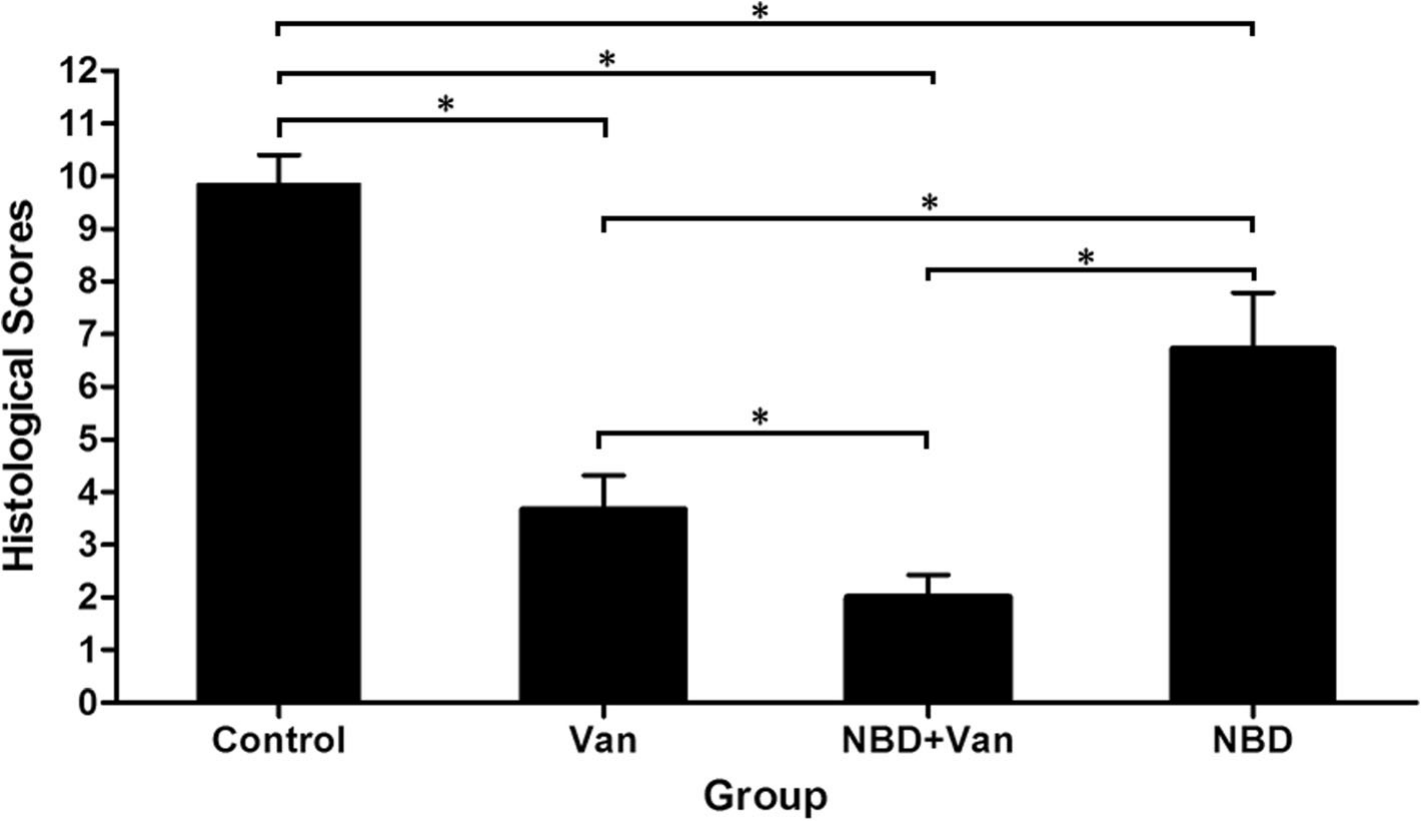
Histological scores in the Control, Van, NBD + Van, and NBD groups 6 weeks after debridement. Results were shown as mean ± standard deviation. Mann-Whitney U-test determined statistical significance where * *P* < 0.05

The varying severity of osteomyelitis in the four groups was further confirmed by histological results (Figs. 7 and 8). Typical signs of osteomyelitis, including acute and chronic intraosseous inflammation, periosteal inflammation, and bone necrosis could be seen in some animals, whereas partial signs were noted in others. A severity score of four or more was found in 7 of 7, 6 of 12, 3 of 12, and 7 of 11 animals in the Control, Van, NBD + Van, and NBD groups, respectively. Histological score of the Control group was significantly higher than that of the Van (*P* = 0.001), NBD + Van (*P* = 0.001), and NBD groups (*P* = 0.038), respectively. The NBD + Van group showed a significantly lower score than the Van (*P* = 0.039) and NBD groups (*P* = 0.01). The Van group also showed a significantly lower score than the NBD group (*P* = 0.047).

## Discussion

The present in-vivo study investigated the effects of NBD peptide on infected bone; it played a key role in inhibiting inflammation and promoting bone remodeling towards osteogenesis, and the effects were better than those produced by antibiotics alone. Despite the failure in demonstrating antibacterial effectiveness of NBD peptide in vivo, antibiotics combined with NBD peptide seemed to be a preferred therapeutic option in chronic osteomyelitis.

In general, a number of research groups have reported the successful treatment of synovial inflammation [19], inflammatory osteolysis [20, 21], inflammatory arthritis [22], cartilage degradation [23], and muscular dystrophy [24] by the use of NBD peptide. Existing rationale for treating chronic inflammatory diseases involving bone resorption by NBD peptide includes the documented reduction of both TNF-α levels and bone destruction based on NF-κB blockade [20]. Furthermore, proinflammatory cytokines, such as TNF-α, are known to be produced in *S. aureus*-induced osteomyelitis [8], which in turn, results in progressive inflammatory destruction of the bone. We aimed to address whether NBD peptide could be made accessible to subjects with osteomyelitis, or whether it could be a therapeutic option in chronic bone infections.

Previous in-vitro studies had shown that a number of bacteria, including *Streptococcus pneumoniae* [25, 26], *Streptococcus pyogenes* [27], and *S. aureus* [28], could activate NF-κB in response to infection. *S. aureus* is the major pathogen among staphylococci and the most common cause of bone infections, which may also activate the NF-κB pathway and ensure that NF-κB activation is required for the phagocytosis of *S. aureus* by macrophages [9]. A recent investigation [29] demonstrated that NF-κB is activated during the development of bone infection. Consistent with this finding, we had shown that selective inhibition of NF-κB, using the NBD peptide, blocks RANKL-induced osteoblast differentiation in vitro [11]. Thus, NF-κB regulates the inflammatory response leading to the development of bone infection, and selective blocking of NF-κB could have therapeutic implication in treating the disease. In this study, our results indicated that specific and selective inhibition of NF-κB by the NBD peptide is an effective approach to treat chronic osteomyelitis. The anti-inflammatory efficacy of NBD peptide in infected bone was assessed by radiologic and histologic means. Extensive chronic osteomyelitis was achieved 3 weeks after infection induction, followed by debridement in all groups, except the Control group. Six weeks after debridement, combination treatment with NBD peptide and vancomycin exhibited the most curative antimicrobial efficacy, with the lowest infection rate, as well as radiological and histological scores. All these values were also lowered in the vancomycin-only group. Meanwhile, local NBD peptide injection alone exhibited infection clearance efficacy in vivo, although it was not comparable to that by vancomycin. Based on these observations, our data may be considered to be in favor of NBD peptide application to chronic osteomyelitis; it exhibited infection elimination efficacy, perhaps owing to the NBD peptide providing an opportunity to selectively abrogate the inflammation-induced activation of NF-κB by targeting the NBD-NEMO interaction.

At present, vancomycin has been introduced in most cases of chronic osteomyelitis treatment [3]. However, it has been losing potency against *S. aureus*, and various forms of resistance to vancomycin have emerged in MRSA. Vancomycin could not cause adequate microbial clearance for treating chronic osteomyelitis induced by MRSA, in this study, and microbiological investigations demonstrated persistence of infection with positive tissue cultures even at study endpoint. Considering the complexity of chronic infections, with antibiotic resistance of MRSA, biofilm formation, low drug penetration, drug activity, and much more, anti-MRSA combinations might be preferable for treating MRSA-osteomyelitis. Compared to vancomycin therapy alone, vancomycin in conjunction with NBD peptide therapy was not expected to elicit a more pronounced antibacterial effect. However, infection clearance rate was enhanced with the combination therapy compared to that in vancomycin alone group. Additionally, infection clearance rate of microbiological examination was increased after NBD peptide therapy compared to that in the untreated control group. Based on these observations, our data are in favor of anti-inflammatory NBD application for treating chronically infected bone, consistent with the previously published in-vitro data [29]. Despite the failure to definitely demonstrate anti-MRSA effectiveness of NBD peptide in vivo*,* there is a possible U-shaped response of bacterial growth to pro-inflammatory cytokines [30], and we speculate that NBD peptide would ameliorate inflammatory responses and may be expected to have a positive influence on host biological response against MRSA infections. Moreover, based on the cellular hysteresis strategy [6], there may be a synergistic effect of vancomycin in conjunction with NBD peptide therapy, which was harnessed to optimize the antibiotic therapy, subsequently achieving enhanced bacterial elimination. To establish these events in vivo, future studies should be conducted to explore inducible physiological effects of NBD peptide, which may, subsequently help to find direct antibacterial effects of NBD peptide for improving antibiotic therapy.

Although temporary inflammation may promote bone healing, persistent and serious infection-associated inflammation could result in suboptimal and impaired bone regeneration [31, 32]. The newly formed bone was quantified by micro-CT, and results showed the volume of newly formed bone in the NBD + vancomycin group to be significantly higher than that in the Van or NBD group; the bone window eventually healed, indicating that NBD peptide could promote bone regeneration effectively only when infection was controlled. We had previously demonstrated that NF-κB activation inhibits osteoblast differentiation induced by TNF-α, and that application of NBD peptide ameliorates this inhibitory effect [11]. Our in-vivo results in the present study showed that NBD peptide is able to effectively ameliorate the inhibition of osteoblast differentiation by chronic inflammation and promote bone regeneration. In conclusion, the NBD peptide not only seemed to prevent bone destruction in chronic osteomyelitis but also accelerated bone regeneration, obliterating the dead space left by debridement.

In the current study, we have utilized an NBD peptide that binds NEMO and compromises the formation of active IKK complex. This sequestration disables IKK functions, primarily the immediate activation of NF-κB. However, physiologically, NF-κB has a crucial anti-apoptotic role in survival, and its pharmacological inhibition may be of limited benefit, owing to the likelihood of generalized apoptosis [20]. In support of this notion, a study reporting the effects of conditional deletion of IKK suggested complete blockage of NF-κB to possibly lead to enhanced apoptosis and damage [33]. Nevertheless, the effects of highly selective pharmacological inhibition of only proinflammatory IKK activity has not yet been fully investigated and reported. This is supported by a number of previous reports [20, 22, 34], showing little or no adverse effect, owing to the blockade of NF-κB, on therapeutic purposes in chronic inflammatory diseases in vivo*.* In previous collagen-induced arthritis experiments, to prevent inflammatory bone destruction [20], NBD peptide was injected into mice, daily for over 4 weeks, without observing any detrimental side effect, or liver or kidney toxicity; injection of the peptide for only the first 5 days was enough to maintain the therapeutic effects for nearly 3 weeks after administration. In our current experiments, we locally injected the rabbits with the NBD peptide, thrice for over 6 weeks, without observing any detrimental side effect or bacterial spread. Local NBD peptide delivery in our experiments minimized the probability of their systemic toxicity caused by systemic usage. However, extensive pharmacological evaluation of this approach would be required to fully determine the effects of long-term use of the NBD peptide to treat chronic osteomyelitis induced by MRSA.

There are several well-established animal models with some kind of implantation that support the establishment of infection and allow the study of chronic osteomyelitis in vivo. However, the implantation of foreign bodies usually does not allow the infection to heal unless the implant is removed. In the present study, we used the model described by Nijhof et al. [16], which aimed to induce infection via the injection of MRSA bacterial suspension without implant infections. The establishment of active infection at 3 weeks, in all animals, could clearly be confirmed by local clinical signs and radiographs. Our data are consistent with the previous finding [35], and demonstrate that a high rate of chronic osteomyelitis could be achieved within 3 weeks of infection induction. The use of sodium morrhuate in the present osteomyelitis model, with the inherent variability of infection intensity, has certain limitations in assessing the effects of NBD peptide in chronic bone infections; however, the model used herein was appropriate to study treatment safety. Clinical assessment and laboratory values were used to study the systemic side effects and bacterial spread after debridement. Neither clinical nor laboratory signs of bacterial spread was observed. We, therefore, concluded that NBD peptide neither worsened bone infections, nor induced bacterial sepsis.

A limitation of the current study was the absence of a group of rabbits with osteomyelitis that received debridement without antibiotics or NBD peptide administration. Therefore, we cannot figure out the specific role of debridement in eliminating infection and stimulating bone regeneration. Another limitation was that a reasonable sample size was not calculated due to lack of a pilot study before the experiment. Our sample size refers to the previous experiments [20, 35], which is more than that in the above reports. Meanwhile, our results have effectively evaluated the anti-inflammatory effect of vancomycin in conjunction with NBD peptide and its effect on promoting bone remodeling in MRSA-induced chronic osteomyelitis. The current results are consistent with our previous investigation, showing that NBD peptide could ameliorate the osteoblast differentiation inhibition caused by NF-κB activation in vitro. These studies may be performed in other laboratories and replicated further using a larger sample size, thereby helping to re-confirm the reliability of our conclusion. Finally, further studies regarding the efficacy of NBD peptides against other types of pathogenic microorganisms, as well as studies considering the combined use of NBD peptides and antibiotics, should be performed in vivo.

## Conclusion

We conclude that NBD peptide induced neither bacterial spread nor worsening of infection. Together with the previous reports of successful and safe treatment of inflammatory bone destruction using NBD peptide, results of the present study are in favor of the applicability of antibiotics combined with NBD peptide to treat osteomyelitis. Since bone defect and residual infection are common problems after debridement, in the treatment of osteomyelitis, despite the failure to definitely demonstrate antibacterial effectiveness of NBD peptide in vivo, antibiotics in conjunction with NBD peptide are suggested to possibly have promising therapeutic potential in osteomyelitis.

## Data Availability

The data can be accessible to the interested researchers by the corresponding authors on reasonable request.

## Abbreviations

BMDs: Bone mineral densities
BV/TV: Bone volume/total volume
IKK: IκB kinase
IL-1: Interleukin 1
IL-6: Interleukin 6
MRSA: Methicillin-resistant *Staphylococcus aureus*
NBD: NEMO-binding domain
NEMO: NF-κB essential modulator
NF-κB: Nuclear factor kappa B
PMMA: Polymethylmethacrylate
RANKL: Nuclear factor kappa B ligand
ROI: Region of interest
*S.aureus*: *Staphylococcus aureus*
TNF-α: Tumour necrosis factor alpha
